# The Role of Inactivation Methods in Shaping Postbiotic Composition and Modulating Bioactivity: A Review

**DOI:** 10.3390/foods14132358

**Published:** 2025-07-02

**Authors:** Ying Zhu, Meiling Xiao, Tangying Kang, Yufeng He, Jiayan Zhang, Yansheng Zhao, Xiang Xiao

**Affiliations:** School of Food and Biological Engineering, Jiangsu University, Zhenjiang 212013, China; ying307@126.com (Y.Z.); 17365227464@163.com (M.X.); 19825266809@163.com (T.K.); hyf_fbs@foxmail.com (Y.H.); jiayanzhang1988@163.com (J.Z.); zhaoys@ujs.edu.cn (Y.Z.)

**Keywords:** postbiotic, probiotic, inactivation, application, bioactivities

## Abstract

Postbiotics, as the metabolic products and cellular components of probiotics, possess the characteristics of being non-living yet retaining biological activity. Postbiotics have unique advantages such as high stability, good security, and a clear target of action. In recent years, they have attracted extensive attention due to their potential roles in immune regulation, anti-inflammation, antioxidation, antibacterial activity, and improving intestinal health. This article systematically reviews the composition of postbiotics and their diversity in fermented foods, with a focus on the impact of different inactivation methods (thermal and non-thermal inactivation) on their biological activities. Many studies have shown that the choice of inactivation method directly affects the immune regulation, anti-inflammatory, and antioxidative functions of postbiotics. Additionally, this review summarizes the application potential of postbiotics in the food industry, the field of medicine and food homology, pet food, and animal breeding, and points out the challenges existing in current research. Future studies need to focus on optimizing inactivation methods to maximize the biological efficacy of postbiotics, thereby promoting the precise application of postbiotics in various fields.

## 1. Introduction

The gut microbiota–host health relationship is a major research focus. Probiotics, defined as “*live microorganisms that, when administered in adequate amounts, confer a health benefit on the host*” [1], have been extensively studied in food science and medicine [2,3,4,5]. Despite their widespread use, limitations persist. Probiotics require intestinal colonization to affect the host’s health through adhesion competition and microbiota modulation [6,7,8,9]. However, the differences in individual intestinal environments and the adaptability of strains lead to probiotics having unstable colonization effects [10], and there are significant variables in their health benefits. Notably, certain probiotics used in food (such as *Enterococcus*) may produce toxins in viable states [11].

These challenges prompted the reevaluation of probiotic mechanisms, revealing that their health-promoting effects might not be entirely dependent on the live bacteria. Subsequent research has demonstrated health-promoting effects from both inactivated bacteria and cellular components/metabolites [12,13,14]. In 2013, Tsilingiri et al. [15] systematically proposed “postbiotics” as bioactive compounds from probiotic metabolites or secretions that can directly or indirectly provide health benefits to the host. In 2021, this definition was refined by the International Scientific Association for Probiotics and Prebiotics (ISAPP) to “*a preparation of inanimate microorganisms and/or their components that confers a health benefit on the host*” [16].

In recent years, postbiotics have received extensive attention due to their potential roles in immune regulation, anti-inflammation, antioxidation, antibacterial activity, and gut health improvement [17,18,19,20,21]. Compared with probiotics, postbiotics have advantages such as high stability, good safety, and multiple action targets [22], and they have shown broad application potential in the food industry, medicinal and edible homology, pet food, and animal breeding fields [23,24]. As a new type of functional component, postbiotics show broad application prospects in promoting host health and physiological regulation. Continued research will further the application value of postbiotics in the health industry [25]. Critically, postbiotic bioactivity depends on preparation processes [26], particularly the inactivation methods that directly affect functional retention and efficacy [27]. Therefore, systematically investigating their inactivation effects is essential for precise applications.

The objectives of this review are to systematically classify postbiotic components and summarize postbiotics in common fermented foods, highlighting their structural diversity and functional synergy. It provides a comparative analysis of thermal and non-thermal inactivation methods and their structure–activity relationships, links treatment parameters with biological activity retention, and evaluates the transformation potential of postbiotics in fields such as food and poultry farming. Meanwhile, challenges such as the lack of clinical evidence and regulatory obstacles are pointed out. These provide a roadmap for optimizing the inactivation strategy and offer a theoretical basis for the scientific research and industrial development of postbiotics.

## 2. Composition of Postbiotics and Postbiotics in Food

### 2.1. Main Components of Postbiotics

Probiotics and synbiotics are commonly utilized as dietary supplements to intervene in the diet and exert health effects on the host by promoting the stability of the gut microbiota [28]. Recognized as one of the most promising microbiota-based interventions, probiotics have significantly advanced animal and clinical research in recent decades, playing a pivotal role in human health maintenance [29,30]. However, the inherent limitations of live bacterial applications have driven researchers to explore alternatives, such as inactivated probiotics. Postbiotics, which exclude viable microorganisms but retain bioactive constituents, can exert beneficial effects on host health through multiple mechanisms [31]. These complex and diverse components are systematically classified into three major categories based on their sources and chemical properties: (1) cellular components, (2) cellular metabolites, and (3) other small bioactive molecules (Table 1).

(1) Cellular components

The cellular components of postbiotics comprise various structural elements, including teichoic acids, peptidoglycan, and surface-layer proteins. Teichoic acid (TA), a distinctive component of Gram-positive bacterial cell walls, constitutes up to 50% of the cell wall mass in many bacterial species. This polymer exists in two primary forms: lipoteichoic acid (LTA) and wall teichoic acid (WTA) [43]. As critical surface molecules of probiotics, teichoic acids exhibit multiple functional properties, demonstrating immunomodulatory, antioxidant, and anti-carcinogenic activities [44]. Notably, WTA serves as a crucial molecular trigger for IL-12 secretion, playing a pivotal role in activating both innate immunity and cellular immune responses [33]. Peptidoglycan (PGN) is an essential macromolecular polymer in bacteria. Soluble peptidoglycan fragments derived from bacteria are regarded as key signaling molecules mediating intraspecific and interspecific communication [45]. In particular, gut microbiota-derived peptidoglycan fragments are increasingly recognized as key effector molecules that impact host biology [46]. Surface-layer proteins (SLPs), located at the interface between the cell wall and membrane, participate in diverse physiological processes. These proteins exhibit multiple bioactive properties, including anti-inflammatory activity, enhancement of epithelial barrier function, and binding capacity for toxic heavy metals [31]. A representative example is the surface-layer protein (B-SLP) from *Lacticaseibacillus paracasei* S-NB, which has been shown to preserve intestinal epithelial barrier integrity in LPS-challenged Caco-2 cell models while simultaneously modulating gut microbiota composition by increasing the relative abundance of beneficial bacterial genera and reducing pathogenic bacteria [47].

(2) Cellular metabolites

Cellular metabolites encompass various components, including extracellular polysaccharides (EPSs), short-chain fatty acids (SCFAs), organic acids, enzymes, vitamins, and bacteriocins. EPSs are natural sugar polymers secreted outside the cells by probiotics during their growth and metabolism. They have diverse functions, and they play crucial roles in immune regulation, anti-inflammation, antioxidation and other aspects [48,49,50]. For instance, intranasal administration of EPSs derived from *Lacticaseibacillus rhamnosus* in mice can downregulate the development of allergic tracheal inflammation and the Th2 cytokine response of sensitized individuals, and increase the total immunoglobulin A(IgA) level, OVA specific and bacterial specific IgA levels, as well as the number of B cells in bronchoalveolar lavage fluid [51]. Short-chain fatty acids (SCFAs), including acetate, propionate, butyrate, isobutyrate, and valerate, are produced through bacterial fermentation of dietary fibers indigestible by the host. SCFAs exhibit diverse bioactive properties and can regulate immunity, maintain intestinal barrier integrity, treat inflammatory bowel disease (IBD), and prevent colorectal cancer. A combination of acetate and propionate has been demonstrated to alleviate inflammation and improve insulin sensitivity in high-fat diet (HFD)-induced diabetic mice, highlighting their metabolic benefits [38].

(3) Other bioactive substances

Beyond intact cellular structures and secreted metabolites, the third critical component of postbiotics encompasses cell lysates—complex mixtures derived from the disruption of microbial cells. These lysates contain both soluble cytoplasmic constituents and insoluble membrane components, representing a holistic repository of microbial biomolecules. Unlike purified metabolites, lysates retain native molecular interactions that may confer synergistic bioactivities.

These bioactive components act synergistically within the host organism, eliciting comprehensive physiological regulation through multi-target and multi-pathway mechanisms. This distinctive characteristic means that postbiotics show unique application value in functional foods, clinical nutritional intervention, and preventive medicine.

### 2.2. Postbiotics in Food

**Figure 1:**
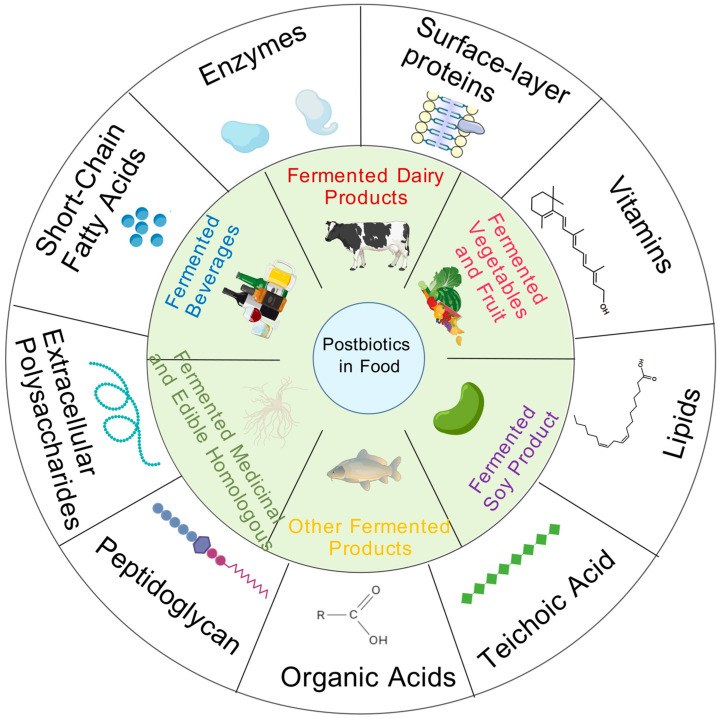
Postbiotic components in common fermented foods [53].

Most fermented foods generally contain microorganisms and bioactive components. These active compounds either occur naturally in the food matrix or are produced by microorganisms during the fermentation process. In addition to active bacteria, non-viable bacteria are also commonly found in fermented foods [52]. These inactive microorganisms and the bioactive substances produced by microbial fermentation together essentially constitute substances with postbiotic characteristics in fermented foods (Figure 1).

(1) Fermented dairy products

Fermented dairy products represent one of the most historically significant and widely consumed functional food categories globally, with a wide variety of varieties, including yogurt, kefir, fermented buttermilk, yogurt wine, traditional milk wine, and various kinds of cheese [54,55]. Recent metabolomic analysis of *Streptococcus thermophilus* S10-fermented brown milk identified 43 differentially abundant metabolites spanning multiple bioactive classes, including peptides, AA, fatty acids and related metabolites, carbohydrate metabolites, vitamins, and nucleosides [56]. These metabolites contribute to the functional characteristics and nutritional quality of the product. Peptides for regulating blood pressure were detected in the mixed milk of skimmed milk and soy milk co-fermented by a commercial starter composed of *Lactobacillus delbrueckii* subsp. bulgaricus CICC 6047 and *Streptococcus thermophilus* CICC 6038 with *Lactiplantibacillus plantarum* Y44 [57]. A total of 26 peptides were detected in the fermented mare’s milk seeds collected from Mongolian Nomads [58]. Relevant studies have reported that the peptides in fermented mare’s milk have the effects of anti-hypertension and anti-diabetes [59,60].

(2) Fermented vegetables and fruits

Fermented vegetables have gained global popularity due to their distinctive flavor profiles, exceptional nutritional value, and demonstrated health benefits [61]. Similarly, fermented fruits are increasingly utilized as functional ingredients in health beverages, serving as lactose-free alternatives for individuals with lactose intolerance [62]. These fermentation processes enhance both the bioactive compound content and sensory characteristics of the final products [63]. Probiotics, especially LAB, produce a variety of metabolites with functional activity. On the one hand, these metabolites shape the unique flavor profile of fermented vegetables, and on the other hand, significantly improve the nutritional value of products [64]^.^ Studies have confirmed that fermented vegetables are rich in vitamins and bioactive extracellular polysaccharide, which confer a variety of health-promoting properties on fermented vegetables, such as anti-obesity, anti-inflammation, immune regulation, and the improvement of the intestinal microecological environment [65,66]. Cucumbers fermented by LAB have certain probiotic potential. *L. brevis* T7 fermentation is friendly to people with lactose intolerance, and *P. parvulus* T13 fermentation has a significant antibacterial effect [67]. Oral administration of fermented red beet extract to Wistar rats could increase the expression of immune cytokines and effectively enhance immunity [68]. In experiments exploring the potential of raw and cooked kimchi as probiotics and postbiotic foods using animal models, it was found that they possess antioxidant and immune-boosting properties regardless of whether they are cooked or not [69]. The Indonesian fermented durian paste called “Tempoyak” has potential health advantages, including immune stimulants, anti-hypercholesterolemia, probiotic effects, preservatives, and antibacterial agents [70].

(3) Fermented soy products

Fermented soy products mainly include fermented bean curd, tempeh, natto, and soy sauce. Fermentation can effectively improve the nutritional quality and functional characteristics of grains [71]. In addition to beans, soybean residue, as processing waste, can produce secondary metabolites, enzymes, and other functional components through microbial fermentation, significantly improving nutrition and sensory perception, and can be transformed into a variety of value-added products [72,73]. Similarly, soybean meal can also be fermented by microorganisms to produce value-added products [74]. After fermentation by *Bacillus subtilis*, the levels of peptides, soluble proteins and free amino acids increased [75]. During the fermentation process, microorganisms utilize the nutrients in the fermentation substrate to grow and reproduce, thereby achieving biological transformation. In the case of natto, a traditional Japanese food, the fermentation process relies on the action of *Bacillus subtilis*. This microorganism can not only secrete protease to degrade soybean protein into biologically active peptides and free amino acids [76], but also synthesize vitamin K2 (methylnaphthoquinone-7), an important fat-soluble vitamin [77].

(4) Fermented beverages

Fermented beverages are liquid products with an alcohol content of less than 1% (by volume), which are made through fermentation by specific microorganisms. Scientific research has confirmed that fermented beverages can enhance the immune function of the body, protect gastrointestinal tissues, control digestive system diseases, and prevent cardiovascular diseases and other health problems [78,79]. Compared with traditional black tea, kombucha contains a large quantity of phenols, vitamins, organic acids and other health-promoting substances. These active components endow kombucha with antioxidant activity, metabolic regulatory functions, and antibacterial effects [80,81]. Makgeolli, made by fermenting various starches with LAB, has antioxidant and lipid-lowering properties and can prevent liver damage to a certain extent [82,83]. The fresh saps of palm trees can be made into light alcohol palm beverages through natural fermentation. During the fermentation process, the content of functional components increases, and the antioxidant activity is higher than that of fresh palm juice [84].

(5) Fermented medicinal and edible homologous plants

Medicinal and edible homologous plants combine the therapeutic effects of drugs with the nutritional value of food [85], and are rich in active compounds [86,87]. These have very great development potential. The method of microbial fermentation can promote the full release of the active ingredients in medicinal and edible homologous plants [88,89] and generate new bioactive components. After fermentation by *Saccharomyces boulardii*, the metabolites of organic acids, flavonoids, and nucleotide derivatives of yam are mainly upregulated [90], and these metabolites are closely related to antioxidant and antibacterial properties. Ginseng, after being fermented with LAB, can be used as a potential dietary nutritional health product to alleviate alcoholic liver injury and performs well in treating antibiotic-associated diarrhea (AAD) symptoms and colonic inflammation [91,92].

(6) Other fermented products

When *Enterococcus rivorum* and *Enterococcus lactis* were used for the mixed fermentation of crucian carp, the protease activity, TCA soluble protein, and flavor substances increased with the increase in fermentation time [93]. In addition to fish meat, when using grass carp bones as the fermentation substrate and fermenting with *Leuconostoc mesenteroides*, the fermentation liquid can promote the growth and development of calcium-deficient rats, which also proves that grass carp bones may be used as a new and highly efficient calcium supplement [94]. Studies have found that the extracellular polysaccharide produced by lactic acid bacteria in the fermentation process can help improve the texture of bread products [95], and these polysaccharides can also form protective biofilms in the human intestine, effectively reducing the damage of external environmental factors on the intestine [96]. Salami is a kind of Western-style fermented sausage. After microbial fermentation, it contains rich short-chain fatty acids [97]. Some volatile substances effectively improve the quality of salami sausages [98], and the production of bacteriocins also makes a great contribution to food preservation [99].

## 3. Classification of Inactivation Methods

From the perspective of production processes, the manufacturing of postbiotics is a systematic biotechnological process, mainly including strain selection, cultivation, fermentation, inactivation, and extraction of active components. The health effects of postbiotics are fundamentally different from those of naturally deceased probiotics, with the core difference lying in whether the specific bioactive factors can be effectively retained during the preparation process.

The significance of the inactivation process is reflected in the following two aspects: ensuring that the final product does not contain live bacteria and determining whether the active factors are retained. Different inactivation processes will directly affect the structural components of cells, and thereby influence the biological activity of postbiotics. During the inactivation process, the residual number of live bacteria can be monitored in real time through cell counting technology, which plays a crucial role in ensuring the thoroughness of inactivation. Under normal circumstances, the plate counting (PC) method can be used to count the cells in the sample. However, in postbiotics, the culture-dependent method cannot be used for counting, and alternative quantitative methods are required instead [100]. Cells can be counted by staining them [101]. Of course, there are other counting methods available for postbiotic cell counting, such as flow cytometry (FCM) [102], real-time PCR (qPCR) [103], and digital PCR (dPCR) [104]. The selection of inactivation methods requires a comprehensive consideration of multiple factors such as postbiotic efficacy, safety, and application scenarios. At present, inactivation technologies can be divided into two major systems: thermal inactivation and non-thermal inactivation. Each method has its own advantages and limitations [105,106]. Thermal inactivation technology inactivates probiotics through heating treatment, and its thermal effect may cause damage to some heat-sensitive active ingredients. In contrast, non-thermal inactivation techniques can better protect thermally unstable components [105,107]. In actual production, the most suitable inactivation scheme should be selected based on the characteristic requirements of the target postbiotics. Through the quantitative analysis of cell count and inactivation efficiency, the corresponding relationship between process parameters and cell inactivation rate can be established. A combined inactivation strategy can be adopted when necessary to balance various technical indicators. At this time, the dynamic monitoring of cell survival rate will provide important data support for process optimization.

### 3.1. Thermal Inactivation

Thermal inactivation technology, as a traditional and efficient inactivation method, holds an important position in the field of probiotic inactivation due to its standardized operation process and controllable production cost. According to different heating methods and conditions, thermal inactivation primarily includes sterilization, pasteurization, and ohmic heating [100]. These heat treatments alter the structure of cell membranes, RNA, DNA, ribosomes, and enzymes, leading to cell death [108]. The thermal inactivation methods of postbiotics are shown in Table 2.

#### 3.1.1. Traditional High-Temperature Sterilization Method

Traditional thermal inactivation technology effectively inactivates probiotics through high-temperature treatment while preserving heat-resistant bioactive components. This method has a simple process and is easy to operate, and is very applicable both in laboratory research and large-scale industrial production. Studies have shown that compared with non-thermal inactivation, high-temperature sterilization has a higher inactivation efficiency. The inactivation rate is as high as 8.18 at 90 °C /10 min, while the inactivation rate is 6.62 after 10 min of treatment at 600 MPa, and only 3.6 after 10 min of ultrasonic treatment at 100% amplitude [122]. The conditions and parameters for sterilization need to be reasonably selected based on the characteristics and expected benefits of different strains, as different sterilization conditions can retain different components with different physiological functions [27,123]. At present, various parameter combinations have been used to inactivate probiotics, such as inactivating *Lactobacillus gasseri* CP2305 at 95 °C/30 s [124], treating *Lactobacillus plantarum* at 90 °C/10 min [122], and treating *Enterococcus faecalis* at 80 °C/30 min [125]. The specific process parameters for thermal inactivation depend on the characteristics of the probiotic strain. When preparing postbiotics, under high-temperature conditions, especially with high pressure as an auxiliary, various heat-resistant microorganisms, including bacterial spores, can be efficiently inactivated, ensuring that the inactivation rate of microorganisms in the product reaches a high level and maintains specific biological activity. The inactivation of *Clostridium perfringens* spores by 600 MPa high-pressure heat treatment (HPTP) at 75 °C was compared with the 75 °C heat treatment alone. The inactivation effect of HPTP was better, and when the temperature of HPTP increased from 38 °C to 75 °C, the spore inactivation was more effective [126]. In addition to temperature, sterilization time is also an important parameter. In a study exploring the impact of thermally inactivated lactic acid bacteria on their competitiveness against pathogenic microorganisms, 80 °C was used for inactivation for 5, 15, and 30 min, respectively. The results showed that the strain inactivated for 15 min could effectively prevent and eliminate the colonization of pathogenic bacteria in the in vitro model of the gastrointestinal tract. It fully demonstrates that the thermal inactivation process can retain and alter certain characteristics of bacterial cells [127]. However, such high-intensity processing conditions also have obvious limitations. High temperatures can cause damage to key postbiotic active substances such as the metabolic products and cell wall components of microorganisms. For instance, it may lead to structural changes in polysaccharides and proteins, affecting their biological activity and functional characteristics. In addition, traditional sterilization methods take a long time to process, consume a lot of energy, and have high requirements for equipment. They need to be equipped with dedicated devices, which increase production costs.

#### 3.1.2. Pasteurization

Pasteurization has a unique advantage in balancing sterilization effect and component retention, as it is a relatively mild thermal inactivation method, which is divided into two processes: low-temperature long time (LTLT) and high-temperature short time (HTST). The low-temperature long-time process usually heats the material to 62–65 °C and maintains it for 30 min. The high-temperature short-time process involves heating the material to 72–75 °C and maintaining it for 15–20 s. The principle is that at a relatively low temperature, through a certain period of heat treatment, the enzyme system and cell membrane structure within the microbial cells are destroyed, thereby achieving the purpose of inactivating the microorganisms, while retaining the nutritional components and flavor substances of the product as much as possible.

In the preparation of postbiotics, the advantage of pasteurization lies in its ability to effectively inactivate most harmful microorganisms and some heat-sensitive beneficial microorganisms, while well preserving some active components of the microorganisms, such as certain biologically active proteins, polypeptides, and polysaccharides, etc. These components may still retain their biological functions after inactivation. The mucin HB05 produced by *Akkermansia muciniphila* can reduce muscle atrophy, enhance muscle strength, and improve muscle health after pasteurization. In another study, pasteurized *Akkermansia muciniphila* MucT (*A. muciniphila*) and its extracellular vesicles (EVs) had a significant preventive effect on obesity [128]. Meanwhile, pasteurized *Akkermansia muciniphila* may treat preeclampsia by improving intestinal barrier function, restoring endothelial function, and regulating metabolic disorders [129]. In addition, the processing temperature of this method is relatively low, which is conducive to maintaining the quality of postbiotic products. Fermented milk was made by co-fermenting pasteurized milk with *Lacticaseibacillus paracasei* PC-01, *Lactiplantibacillus plantarun* Lp-6, *Lactobacillus helveticus* H9, and *Bifidobacterium animalis* subsp. lactis Probio-M8. After pasteurization (75 °C/25 s), human intervention was carried out. The results showed that the intake of pasteurized fermented milk alleviated sub-health symptoms, mainly affecting the intestinal microbiome, metabolome, and serum metabolites [130]. However, due to the relatively low processing temperature of pasteurization, it is difficult to completely inactivate some heat-resistant microorganisms and spores, resulting in a relatively short shelf life for the product. Moreover, it needs to be combined with storage conditions such as refrigeration to prevent the inactivated microorganisms from regrowing and reproducing under suitable conditions.

#### 3.1.3. Ohmic Heating

Ohmic heating (OH) is a process of heat transfer that relies on electrical conductivity for heating. It can rapidly and evenly heat the medium. When alternating current passes through microorganisms, internal heat is generated due to resistance, and electrical energy is converted into thermal energy, thereby achieving the purpose of cell death [131,132]. The electric field strength is considered one of the most important process parameters for microbial inactivation. Studies have shown that for *Lactobacillus acidophilus* LA 05, *Lactobacillus casei* 01, and *Bifidobacterium animalis* Bb 12, applying 4, 8, and 12 V/cm at 95 °C and 60 Hz for 5–7 min can completely inactivate probiotic cultures. Flow cytometry and scanning electron microscopy observations indicated that 8 V/cm caused less cellular damage compared with the other voltages, making it the recommended voltage for ohmic heating [132]. Other thermal inactivation methods, such as infrared heating, microwave heating, and radiofrequency heating, are less commonly used in postbiotic preparation [133].

### 3.2. Non-Thermal Inactivation

Non-thermal inactivation deactivates probiotics through physical or chemical methods, preserving heat-sensitive components. Compared with thermal inactivation, non-thermal technologies have gained increasing attention due to their ability to better retain the bioactivity of heat-sensitive components [105]. Non-thermal inactivation physically or chemically disrupts the cellular structure of probiotics, damaging cell membranes, enzymes, or DNA to inactivate the microorganism, while maximizing the functionality of metabolites and cell components [134]. Common non-thermal inactivation methods used in postbiotic preparation include ultraviolet (UV) inactivation, high-pressure inactivation, ultrasonic sterilization, pulsed electric fields (PEF), irradiation, supercritical carbon dioxide (sCO_2_), and extreme pH [100]. The non-thermal inactivation methods of postbiotics are shown in Table 3.

#### 3.2.1. Ultraviolet Inactivation

Ultraviolet (UV) radiation is a non-ionizing radiation with germicidal properties. UV inactivation utilizes ultraviolet light to destroy the DNA of probiotics, causing them to lose their reproductive ability [140]. Meanwhile, lipid- and protein-based biomolecules are also important targets of ultraviolet light [141]. After ultraviolet irradiation, reactive oxygen species (ROS) are produced, causing oxidative damage to microbial cells, which also helps to enhance the inactivation effect of microorganisms. Ultraviolet sterilization is usually used to kill microorganisms in air or water, but it is less frequently applied in the preparation of postbiotics. It is suitable for the preparation of postbiotic components that are sensitive to heat. The details of inactivating probiotics by ultraviolet light are not described in detail. Generally, UV inactivation is performed under a 39 W UV lamp for 5–30 min [142]. However, the effectiveness of UV inactivation is influenced by the concentration and transparency of the bacterial suspension, and it may not completely inactivate probiotics in high-density cultures [105]. In practical applications, combining ultraviolet inactivation with other methods can achieve better inactivation effects.

#### 3.2.2. High-Pressure Inactivation

High-pressure inactivation is a method that involves subjecting the material to a pressure of 400–600 MPa at 37 °C for 10 min. This process disrupts the cell membranes of probiotics, causes protein denaturation, and reduces the intracellular pH. It has the advantages of efficient inactivation and retention of heat-sensitive components, but the equipment cost is relatively high and it is suitable for small-scale production [142]. When the high-pressure inactivation process is used alone, a relatively long processing time is required. However, if high pressure is combined with heat treatment or homogenization treatment, it is more likely to cause changes in the protein structure, thereby achieving a better inactivation effect [143,144,145]. Compared with other inactivation methods, using high-pressure treatment as a probiotic inactivation method may have an impact on cell integrity.

#### 3.2.3. Ultrasonic Sterilization

Ultrasound is defined as sound waves with frequencies above the human auditory threshold (>16 kHz) [146]. Ultrasonic treatment generates sound waves and related mechanical vibrations, causing alternating expansion and compression cycles in the medium. During the expansion stage, high-intensity ultrasonic waves generate small bubbles one by one. When the bubbles reach the volume threshold volume, they burst violently. The process of bubble formation, expansion, and explosion is called cavitation. The principle of ultrasonic sterilization is to utilize the cavitation effect generated by ultrasonic waves. When bubbles explode, they release energy, causing both temperature and pressure to rise, thereby destroying the cell structure, leading to cell wall rupture, cell membrane disturbance and thinning, as well as DNA damage [147,148]. Ultrasonic waves, as a non-thermal processing technology, are widely used in the inactivation of microorganisms and enzymes [149]. This method is simple to operate and suitable for laboratory-scale applications, but its large-scale application is limited, and it may generate free radicals that affect the stability of postbiotics. In practical applications, the use of ultrasonic sterilization alone may not achieve the expected fire-extinguishing effect. It can also be combined with other methods, such as ultrasonic and ultraviolet, as an effective and novel non-thermal treatment method, which is a promising alternative to thermal sterilization in the juice industry [150].

#### 3.2.4. Pulsed Electric Fields Inactivation

Pulsed electric field (PEF) is a sustainable technology that can apply a high-intensity electric field and extremely short voltage pulses to a sample placed between two electrodes [151]. When the voltage and related electric field are higher than the critical transmembrane potential, it can be effectively used to inactivate microorganisms. Under such circumstances, the biological cells will undergo electroporation, inducing a stress response and generating secondary metabolites under stimulation [152]. Different microorganisms have different resistances to pulses. When four types of microorganisms, namely *Escherichia coli*, *Listeria monocytogenes*, *Lactobacillus plantarum*, and *Saccharomyces cerevisiae*, were treated with PEF simultaneously, *Listeria* was the most resistant strain under PEF treatment [153]. Therefore, to achieve the goal of complete inactivation, when choosing PEF for sterilization, it is necessary to fully consider the probiotic strains to regulate different electric field intensities and specific energy parameters [154]. PEF has become a widely used inactivation method in the dairy industry. The use of PEF at 10–24 kV/cm and 110–115 kJ/L can inactivate *Lactobacillus plantarum* ATCC 8014 and *Lactobacillus plantarum* WCFS1 in oat milk, proving that PEF can be a possible alternative to thermal pasteurization for the sterilization of plant-based dairy products [155].

#### 3.2.5. Other Non-Thermal Inactivation

Irradiation sterilization is a new non-thermal sterilization technology that exposes microorganisms or food to ionizing radiation emitted by radioactive isotopes such as 137 cesium or 60 cobalt. Microbial cells absorb radiation, causing damage to their DNA. Different microbial strains have different resistance to radiation [100].

For postbiotic production, supercritical carbon dioxide (sCO_2_) is a carbon dioxide fluid maintained above critical temperature and pressure (supercritical fluid). At 31 °C and 7.3 MPa, carbon dioxide exists in a supercritical state. Supercritical carbon dioxide can remove important components of the cell membrane, achieving the inactivation of probiotics [156]. Combining supercritical carbon dioxide with high pressure to treat microorganisms can achieve a better inactivation effect, and pressure is also an important factor affecting the inactivation effect [157].

pH can change the structure of the cell membrane and cause protons to flow out of the cell, so adjusting the pH can also lead to the inactivation of probiotics [27]. Some strains have acid resistance, and the pH and processing time should be set according to the characteristics of the strain when preparing postbiotics [158].

## 4. Changes in Postbiotic Bioactivity Under Different Inactivation Methods

**Figure 2:**
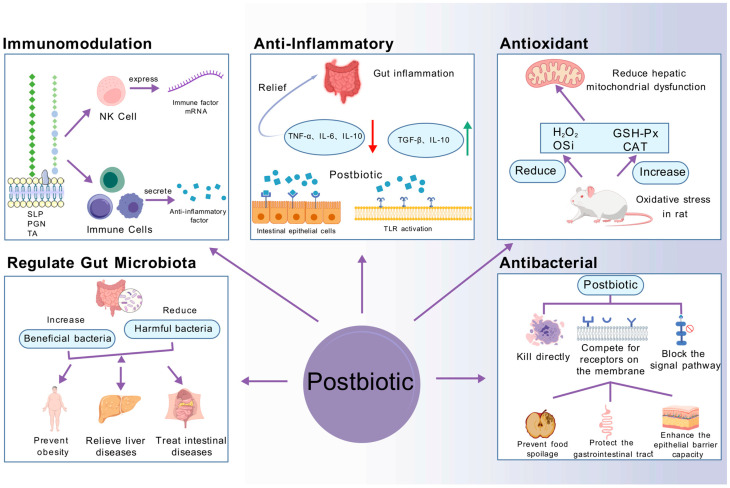
The biological activities of postbiotics after different inactivation methods [53]. (1) Immunomodulation—postbiotic components act on immune cells, achieving immune regulation by enhancing their activity and stimulating the production of immune factors. (2) Anti-Inflammatory—postbiotic components act on immune cells, achieving immune regulation by enhancing their activity and stimulating the production of immune factors (the red arrow represents a decrease, and the green arrow represents an increase). (3) Antioxidant—reduce the levels of Osi and H_2_O_2_ and increase the activities of glutathione peroxidase and catalase in the liver in oxidative stress in rats. (4) Antibacterial—postbiotics achieve antibacterial effects by directly killing pathogenic bacteria, competing for receptors on the membrane, and blocking signaling pathways. (5) Regulate Gut Microbiota—inactivated probiotics regulate immunity and inflammation by inhibiting the growth of harmful bacteria in the intestines and promoting the reproduction of beneficial bacteria, preventing obesity, relieving liver diseases, and regulating intestinal health.

Postbiotic components, such as cell surface substances, peptidoglycan, teichoic acid, lipoteichoic acid, and whole cells, exhibit varying bioactivity depending on the strain and inactivation method. Different inactivation methods have distinct effects on the bioactivity of postbiotics, including immunomodulation, anti-inflammatory, antioxidant, and antimicrobial activities (Figure 2).

### 4.1. Immunomodulation

Dysregulation of immunomodulation can lead to various diseases, such as allergies, cancer, liver disease, diabetes, lupus erythematosus, and myasthenia gravis [159]. Studies have shown that postbiotics positively promote immunomodulation [105,160], effectively improving lymphocyte proliferation, enhancing macrophage function, and increasing NK cell activity, thereby regulating immune function [161].

Since cell components such as surface proteins, teichoic acid, and peptidoglycan can be affected by inactivation methods and strain differences, postbiotics will also have differences in their role in immune regulation [162]. After inactivating *Lactobacillus gasseri* OLL2809 by γ irradiation (2800 Gy), cell-free supernatant (sup) was collected. The surface-layer proteins (SLPs) contained in the sup have potential immunomodulatory abilities against mouse bone marine-derived dendritic cells (DCs) and can stimulate the expression of specific cytokines in iDCS [163]. The intact cell wall (ICW) of thermally inactivated *L. plantarum* containing WTA can induce macrophages to produce IL-12 through macrophages via actin-dependent phagocytosis rather than the TLR2 signaling axis pathway [33]. Oral administration of *Lacticaseibacillus rhamnosus* GG LTA (LGG-LTA) can activate mouse DC and T cells, increase the activation ability of T cells, promote the maturation and proliferation of DC cells, and reduce the immunosuppression induced by UVB and the development of skin tumors in mice [164]. The whole peptidoglycan (WPG) extracted from *Lactobacillus paracasei* subsp. paracasei M5 strain after ultrasonic treatment exerted cytotoxic effects on colon cancer HT-29 cells in a dose-dependent manner, upregulating pro-apoptotic genes and downregulating anti-apoptotic genes simultaneously [165]. Heat-inactivated *Lactobacillus gasseri* TMC0356 activates TLR signaling pathways, promoting macrophage secretion of cytokines such as IL-12 and TNF-α [162,166]. Heat-inactivated cells show increased resistance to N-acetylmuramidase, indicating intact cell wall structures post-inactivation [162]. Oral administration of heat-inactivated *L. gasseri* TMC0356 significantly increases spleen NK cell activity and lung cytokine (e.g., IL-2 and IFN-α/β receptor 1) mRNA expression in senescence-accelerated mice (SAMP1), indicating enhanced cell-mediated immunity [161]. Higher temperatures (90 °C) for shorter durations (5 min) better retain postbiotic immunomodulatory activity compared with lower temperatures (70 °C) for longer durations (30 min). Raz et al. [167] found that radiation-inactivated paraprobiotics (using cesium-137 at 8 Gy/min overnight) were more effective in treating colitis in animal models than heat-inactivated (100 °C/30 min) paraprobiotics.

### 4.2. Anti-Inflammatory

Inflammation, commonly referred to as “swelling”, is the defense response of tissues in the body after an irritating trauma [168], characterized by redness, swelling, heat, pain, and dysfunction.

Probiotics can regulate inflammatory responses in multiple ways [169]. With the deepening of research, more and more evidence indicates that the anti-inflammatory effect is mainly attributed to the bacterial components and metabolites of probiotics [170]. The short-chain fatty acids produced by probiotics can indirectly regulate inflammatory responses by binding to specific receptors in intestinal epithelial cells [171]. Butyrate enhances anti-inflammatory effects by promoting tight junctions of immature intestinal epithelial cells and the transcription of mucus genes [172]. After 30 min of ultrasound treatment, LTAs derived from *Lactiplantibacillus plantarum* A3, *Limosilactobacillus reuteri* DSMZ 8533, and *Lactobacillus acidophilus* CICC 6074 can downregulate the expression of pro-inflammatory cytokines (TNF-α, IL-6) and anti-inflammatory cytokine (IL-10), significantly alleviating inflammatory responses. Notably, the LTA from *L. reuteri* was found to inhibit LPS-induced activation of the MAPK and NF-κB signaling pathways [135]. PGN obtained by boiling *Lactobacillus acidophilus* bacterial cells in 4% SDS for 30 min and then ultrasonic treatment had good anti-inflammatory ability in model RAW 264.7 cells, and the levels of inducible NO synthase (iNOS) and cyclooxygenase-2 (COX-2) were significantly decreased [173]. In the heat-inactivation method, some heat-resistant anti-inflammatory components are retained and continue to exert biological effects. Heat-inactivated *Streptococcus thermophilus* ST7 exhibits significant anti-inflammatory activity in mouse models, alleviating TLR3 agonist (poly I:C)-induced intestinal inflammation, inhibiting pro-inflammatory factors (such as IL-6, TNF-α), and promoting anti-inflammatory factors IFN-γ expression [174]. The cell wall components of bacteria (peptidoglycan, lipophosphatidic acid) still exert anti-inflammatory effects by regulating the TLR signaling pathway after thermal inactivation [105]. In Jhong et al.’s study [175], heat-inactivated *Lacticaseibacillus paracasei* GMNL-653 exhibited anti-osteoporotic and anti-inflammatory effects in ovariectomized mice by restoring gut microbiota dysbiosis, reducing pro-inflammatory factors (such as IL-17, LPS), and increasing anti-inflammatory factors (such as TGF-β, IL-10). Extracellular vesicles (LP-UJS-EVs) produced by *Lactobacillus plantarum* UJS001 (LP-UJS) (LP-UJS) induce M2 polarization of macrophages, promote the release of IL-10 and TGF-β, and inhibit the release of histamine, IL-6, and TNF-α. It also reduces the abundance of *Coprococcus*, *Parabacteroides*, *Staphylococcus*, and *Allobaculum*, and increases the abundance of *Prevotella*, playing a key role in restoring the gut barrier and alleviating gut inflammation [176].

### 4.3. Antioxidation

Studies have shown that the excessive production of free radicals in the body may accelerate aging, reduce basal metabolic rate, and impair toxin excretion, potentially increasing the risk of diseases such as cancer and gut disorders [177]. Therefore, finding antioxidants or agents that slow oxidative reactions is crucial.

Research has shown that consuming probiotics or probiotic supplements can improve the body’s antioxidant capacity [178,179]. As research on postbiotic functional activity continues, it has been found that postbiotics also enhance the body’s antioxidant capacity. Specifically, postbiotics produced by *Lactiplantibacillus plantarum* are a natural source of antioxidants [180]. Different inactivation methods may impact the antioxidant capacity of postbiotics. In Sun et al.’s study [26], *Lactobacillus paracasei* ET-22 and *Bifidobacterium lactis* BL-99 were subjected to heat treatment at 70–121 °C for 10 min, disrupting cell structures to fully release cellular contents. At temperatures below 100 °C, the antioxidant properties of ET-22 and BL-99 postbiotics were unaffected by temperature and heating. However, at 121 °C, the chemical properties of postbiotics were significantly altered, with several bioactive components (such as zomepirac, flumethasone, 6-hydroxyhexanoic acid, and phenyllactic acid) showing reduced bioactivity, leading to decreased antioxidant activity. Guerrero-Encinas et al. [181] obtained cell contents of *Lacticaseibacillus casei* CRL-431 through enzymatic and ultrasonic treatment and established an aflatoxin B1-induced oxidative stress rat model. In vitro digestion tests showed that postbiotics significantly reduced the oxidative stress index (OSi) and increased liver glutathione peroxidase and catalase activity, with lower H_2_O_2_ levels demonstrating the antioxidant effects of *L. casei* CRL-431 cell contents. After inactivating the bacterial suspension of *Lacticaseibacillus paracasei* for 15 min at 121 °C, the *Lacticaseibacillus paracasei* Postbiotic-P6 obtained by ice water bath ultrasound had outstanding antioxidant performance, and the reducing ability increased from 0.279 to 2.322 at a concentration of 1.25–12.5%. The scavenging rate of DPPH radical scavenging activity at a concentration of 2.5–50% was 38.9–92.4%, and that of hydroxyl radical scavenging activity at a concentration of 0.5–4% was 4.66–10.38% [182].

### 4.4. Antibacterial Properties

The presence of bacteria poses a threat to human health, driving the search for new antimicrobial substances. Although research on postbiotics is relatively recent, their excellent antimicrobial properties have been discovered [183]. *L. plantarum* can produce a large amount of antibacterial metabolites during its growth process and is regarded as the most promising strain for controlling bacterial growth and generating bacteriocins [184]. Studies have shown that postbiotics can resist pathogenic bacteria and spoilage bacteria, preventing infectious diseases and food spoilage, inhibiting pathogen colonization in the gut, and preventing induced gut diseases [185].

In Ishikawa et al.’s study [186], heat-inactivated (121 °C/30 min) *Lactobacillus plantarum* b240 postbiotics inhibited bacterial adhesion and invasion in vitro, interfering with *Salmonella typhimurium* binding to cell surface receptors, thereby reducing bacterial adhesion and effectively inhibiting *S. typhimurium* invasion of cells, preventing bacterial entry and spread in the body. Nakamura et al. [187] screened *Leuconostoc mesenteroides* 1RM3 from fermented fish, which, when heat-inactivated, prevented *Listeria monocytogenes* gastrointestinal invasion and infection. In vitro experiments showed that heat-inactivated *L. mesenteroides* 1RM3 bacterial cells or metabolites interacted with Caco-2 cells, altering cell surface structures or signaling pathways, hindering *L. monocytogenes* adhesion and invasion. Similarly, in animal experiments, heat-inactivated *L. mesenteroides* 1RM3 prevented bacterial invasion by modulating gut microbiota balance, enhancing gut barrier function, or activating immune responses. Other postbiotics can also be used as antimicrobial agents in food [183]. Unsterilized rapeseed meal was mixed and fermented with *Bacillus subtilis*, *Pediococcus acidilactici*, and *Candida tropicalis*. On the first day of fermentation, *C. tropicalis*, and, on the second day, *B. subtilis* increased the content of polypeptides, and the microbial diversity significantly decreased after fermentation, indicating that mixed bacterial fermentation can inhibit the growth of miscellaneous bacteria [188].

### 4.5. Regulate Gut Microbiota

Gut microbiota are normal microorganisms in the human gut, playing important roles in metabolism, nutrition, physiology, and immunity [139]. Maintaining a good gut microbiota may help regulate immune responses and reduce inflammation [2,189]. The homeostasis of gut microbiota affects various physiological and biochemical functions [190], leading to various diseases.

Studies have shown that probiotics can improve gut microbiota balance [191], and postbiotics have similar effects. In Kimoto-Nira et al.’s study [192], heat-inactivated *Lactococcus lactis* G50 affected gut immunity and microbiota in senescence-accelerated mice (SAMP6). For beneficial and common gut bacteria, heat-inactivated *L. lactis* G50 could not proliferate in the gut or produce antimicrobial substances such as lactic acid and bacteriocins to influence other microorganisms’ growth, thus having no significant regulatory effect on these gut microbes. However, for harmful bacteria (H2S-producing bacteria), heat-inactivated *L. lactis* G50 inhibited their growth, possibly by adhering to gut cells or co-aggregating with pathogens, preventing their colonization and growth. Heat-inactivated *Levilactobacillus brevis* PDD-2 significantly increased the relative abundance of *Erysipelotrichaceae* in the gut, better promoted oxidative stress balance in the liver, and effectively alleviated chronic alcoholic liver disease through the gut–liver axis [193]. As an important part of metabolic activities, the imbalance of the gut microbiota can affect physical health [194]. Obesity is a typical example caused by the imbalance of the intestinal microbiota, and obesity can lead to various metabolic diseases, including non-alcoholic fatty liver disease and diabetes [195]. The connection between obesity and the intestinal microbiota is close, and there is a causal relationship [196,197]. For the treatment and prevention of obesity, the gut microbiota is a good entry point. *Lactiplantibacillus plantarum* J26, which was inactivated by ultrasound at 400 W/ 30 min and pasteurization, intervened in the composition of the intestinal microbiota of mice, increased the abundance of *Lactobacillus* and *Bifidobacterium*, reduced the abundance of *Faecalibacterium* and *Erysipelotrichaceae* related to obesity, improved obesity, and reduced liver tissue damage at the same time [198].

## 5. The Application of Postbiotics

**Figure 3:**
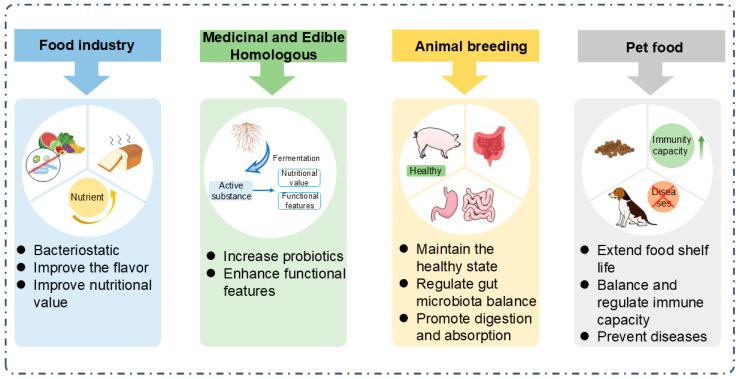
The application of postbiotics in different fields.

Postbiotics, due to their unique probiotic functions, are often used as food nutritional supplements or biological preservatives in the food industry and have high application value. Beyond traditional food applications, postbiotics also show promising market potential across diverse industries, including medicine and food homology, and animal feed (Figure 3).

### 5.1. Food Industry

With the continuous improvement of consumers’ health awareness, the food industry is facing an urgent need to develop new food ingredients that are functional, safe, and stable. Postbiotics are becoming an important direction for food industry innovation due to their unique health-promoting properties and processing stability. LAB fermentation can not only produce rich organic acids, bactericins, and extracellular polysaccharides and other active substances, which can be used as natural preservatives or flavor substances in fermented foods, dairy products, etc., but also improve the physical and chemical properties of these products and increase their digestibility [199].

Studies have shown that the cell-free supernatant (CFS) of *Lactobacillus pentosus* 86 has a significant inhibitory effect on the pathogenic fungus *Alternata* in fruits and vegetables. Even under thermal treatment (40–100 °C), different protease solutions and acidic conditions (pH 2–4), the inhibitory activity of CFS can reach up to 80% [200]. Parvarei et al., produced yogurt using Lactobacillus acidophilus ATCC SD 5221, Bifidobacterium lactate BB-12, and inactivated probiotics. After comparing various indicators, it was found that inactivated probiotics could be used as a suitable substitute for live bacteria in functional yogurt [201]. Adding heat-inactivated *Lactobacillus casei* CP2305 to beverages and conducting intervention on constipated people, it was found that the postbiotic-containing beverages promoted the increase of SCFA concentration in the intestines of the subjects, changed the composition of intestinal microbiota, and effectively improved the constipation symptoms of the subjects [202]. The extracellular polysaccharides of *Lactobacillus plantarum* added to cheese can improve the appearance and overall quality of cheese, effectively compensating for sensory defects. When added to sourdough and wheat bread, it can improve the hardness, color, and moisture content of bread [203,204]. A strain of *Leuconostoc mesenteroides* XR1 that produces exopolysaccharides (EPSs) was isolated from Xinjiang Kefir grains. XR1-EPS can endow fermented milk with higher viscosity and elasticity and can be used as a natural organic additive to replace the chemical additives in dairy products [205]. Postbiotics show broad prospects in the food industry, but their application still faces several challenges. Postbiotics is an extremely complex system, and there is a lack of basic research support from the perspective of food applications. The efficacy results of postbiotics are mostly based on cell experiments and animal experiments, and there is less evidence from human experiments, and even less from clinical trials. Domestic and foreign regulatory agencies have not established a regulatory or standard management framework for postbiotic foods or dietary supplements, and there are no clear regulations on the use of postbiotics and related requirements.

### 5.2. Medicinal and Edible Homologous

Medicinal and edible homologous plants, such as yam, poria, wolfberry, tangerine peel, and hawthorn, are rich in active components such as polysaccharides, flavonoids, and saponins. Microbial fermentation can enhance their biological efficacy, especially in terms of lipid reduction, anti-obesity, and antioxidation, where the effects have been significantly improved.

Monacolin K (MK), a secondary metabolite produced by *Monascus*, has lipid-lowering effects. *Monascus* is fermented with quinoa, which has high nutritional value, to produce *Monascus*-fermented quinoa (MFQ). MFQ may regulate the amino acid levels of hyperlipidemic mice by influencing metabolic pathways such as phenylalanine, tyrosine, and tryptophan metabolism. It alleviated hyperlipidemia induced by a high-fat diet [206]. Gastrodia elata Bl. (GE) is a food of medicine and food homology which can alleviate decline in memory and learning ability. Fermenting GE with *Lactobacillus plantarum*, *Acetobacter pasteurianus*, and *Saccharomyces* (FGE) can enhance the richness of the intestinal microbiota, especially beneficial bacteria, and improve the symptoms of Alzheimer’s disease (AD) by regulating the amino acid metabolic pathway [207]. Although the application of postbiotics in the field of medicinal and edible homologous has broad prospects, it also faces multiple challenges, involving multiple links such as technology, standards, markets, and scientific research. The selection of strains and fermentation processes need to be standardized, and the identification of postbiotics, efficacy, and safety need to be verified in multiple aspects.

### 5.3. Animal Breeding

In the field of animal breeding, there are many studies on active probiotics, while the research on inactivated probiotics is still in its infancy. The application of postbiotics in animal breeding mainly involves adding postbiotic components to feed. Compared with ordinary feed, the quality and bioactive compounds of feed containing postbiotic components are effectively retained [208]. Heat stress will lead to bacterial infection and inflammation in poultry. The antibacterial components in epigenome can regulate the gut microflora of poultry breeding, improve the integrity of the intestinal mucosal barrier, and thus maintain the intestinal health of poultry and improve the growth performance of poultry.

The addition of compound probiotics (BFI) composed of heat-inactivated *Bacillus subtilis* and *Lactobacillus acidophilus* to the diets of yellow feather broilers not only improved feed efficiency, but also reduced the plasma cholesterol content and creatinine content of broilers [209]. When the cell-free supernatant of *Lactobacillus plantarum* RI11 was used as a dietary supplement for broilers at an addition amount of 0.6%, it could upregulate the mRNA expressions of IL-10, ileumzonula occludens-1, and andmucin 2, and downregulate the levels of IL-8, tumor necrosis factor α, heat shock protein 70, and α -1-acid glycoprotein [210]. Izuddin et al., found that after adding 0.9% cell-free supernatant (CFS) of *Lactobacillus vegetarum* RG 14 to their diet, both the height and width of the rumen papilla in lambs increased, the contents of white blood cells, lymphocytes, basophils, neutrophils, and platelets in the blood decreased, and the expression of IL-6 mRNA in the jejunum increased. The expressions of IL-1β, IL-10, and TNF mRNA decreased. Probiotics also upregulated the expressions of TJP-1, CLDN-1, and CLDN-4 mRNA, and the integrity of the intestinal barrier was improved [211]. In addition to directly adding postbiotics to the feed, some grains that have been fermented by probiotics can also be added. These fermented grains also contain postbiotic components, and these components can also play health roles such as anti-obesity in the feed [212,213]. There are still great challenges in the application of postbiotics in animal breeding. The effective components, dosage, and specification of postbiotic need to be further studied. There is a lack of unified evaluation criteria for the efficacy of epigenetic elements in different strains, and there are few studies on the mechanism of epigenetic elements and the connection with their signaling pathways. With the increase of research data on epigenetic elements in animals, the application of epigenetic elements in animal breeding will be more extensive.

### 5.4. Pet Food

With the significant improvement of the public’s material living standards, the scale of pet breeding has also increased significantly, but the outbreak rate of pet diseases has also increased significantly, so the development of green, safe, and healthy pet feed has become the focus of the pet industry. By adding an appropriate amount of epigenesis to pet diets, it can not only better regulate the gut microbiota of pets, balance and regulate the immune capacity of the body, effectively inhibit the reproduction and growth of pathogenic microorganisms, but also extend the shelf life of products. The most common main ingredient in pet feed on the market is grains. Grains are prone to contamination by mycotoxins. Aflatoxin is mainly a mycotoxin produced by Aspergillus flavus. This mycotoxin directly affects pets by contaminating feed [214], seriously threatening the safety of pet feed [215,216].

Studies have shown that the CFS and non-living cells of *Bacillus plantarum* 83114 significantly inhibit fungal growth, with a growth rate of zero [217]. Using the fermentation products of *Lactobacillus fermentum* and *Lactobacillus defolius* (LBFP) as dietary supplements for Beagle dogs, research has found that LBFP alters the fecal microbiota and regulates oxidative stress markers after transport stress, preventing oxidative damage to benefit dogs [218]. Elderly dogs will suffer from various diseases due to decreased immune function. Usually, vaccination can be used to prevent dogs from getting sick. However, compared with mature dogs, the antibody titer induced by neoantigens in elderly dogs is significantly decreased, and the preventive effect on diseases is not good [219]. Wambacq et al. [220] added short-chain fructo-oligosaccharides (scFOS) and post-yeast biocombination (scFOS+) to the diet of elderly dogs and found that the addition of scFOS+ could effectively improve the immunosenescence of elderly dogs and promote their immune response. Transportation is a common phenomenon in the daily pet-raising process. Due to the influence of factors such as turbulence, noise, vibration, and ambient temperature, pets are prone to stress reactions that harm their health. Feeding yeast fermentation products (SCFP) during transport can keep the concentration of markers of oxidative stress stable in pointer dogs and reduce stress response by inhibiting the activation of innate immune cells [221].

## 6. Conclusions

Postbiotics, functional derivatives of probiotics, are characterized by high stability, safety, and multi-target efficacy. This review systematically outlines the composition and diversity of postbiotics in fermented foods, emphasizing the critical role of inactivation methods (thermal and non-thermal) in modulating their bioactivities, including immunomodulation, anti-inflammatory, antioxidant, and antimicrobial effects. The key findings highlight that while thermal inactivation is efficient and scalable, it may compromise heat-sensitive components, whereas non-thermal methods better preserve functionality but face challenges such as high costs and technical complexity. Despite their potential in food, medicine, and animal feed, postbiotic applications are hindered by insufficient clinical validation, a lack of standardized regulatory frameworks, and limited understanding of mechanistic pathways. Future research should prioritize optimizing inactivation techniques to balance efficacy and stability, expanding human trials to validate health claims, and employing multi-omics technologies to elucidate the precise mechanisms of action. Addressing these challenges will unlock the full potential of postbiotics as safe, stable, and multifunctional alternatives to traditional therapies.

## Figures and Tables

**Table 1:** Composition and functions of postbiotics.

Source	Component	Specific Component	Function	Reference
Cellular components	Teichoic acid (TA)	Lipoteichoic acid (LTA)	Regulated inflammatory factors TNF-α and IL-10 levels.	[32]
		Wall teichoic acid (WTA)	Stimulated macrophages and dendritic cells to secrete IL-12, modulating immune responses.	[33]
	Peptidoglycan (PGN)	Cell wall components	Intervention regulated the functions of T lymphocytes and dendritic cells, alleviating Salmonella-induced inflammation in mice.	[34]
	Surface-layer proteins (SLPs)	Cell surface protein	Reduced adhesion of *E. coli* and *Salmonella* to HT-29 cells and inhibited pathogen-induced apoptosis.	[35]
Metabolites	Extracellular polysaccharide (EPS)	Probiotic-secreted polysaccharides	Decreased NO production and reduced expression of the pro-inflammatory cytokine IL-6.	[36]
	Short-chain fatty acids (SCFAs)	Propionate and butyrate	Inhibited the production of inflammatory factors such as IL-6 and IL-4, reduced reactive oxygen species (ROS) expression, and enhanced IL-10 and IFN-γ expression, alleviated cellular inflammation.	[37]
	Organic acids	Lactic acid and acetic acid	Reduced HFD-induced inflammation and improved insulin sensitivity in diabetic mice.	[38]
	Enzymes	Digestive enzymes, synthetases, and transferases	The fermentation of soybean residue by *Bacillus* natto can produce nattokinase and α-amylase, which have the effects of lowering blood sugar and blood pressure.	[39]
	Vitamin	B vitamin	Under the condition of pH 2–4, the mycelia growth of Streptospora Geisenensis was inhibited, while antifungal activity was maintained up to 80%.	[40]
	Bacteriocins	Ribosomally synthesized antimicrobial peptides	Involved in cell signaling, possesses antimicrobial activity, and inhibits pathogen growth.	[41]
Other bioactive substances	Bacterial lysates	Bioactive factors released by bacterial cell lysis	Reduced periodontitis and dental caries by modulating signaling pathways, which can help improving oral health.	[42]

**Table 2:** Examples of thermal inactivation of postbiotics.

Strain	Inactivation Method	Conditions	Target	Function	Reference
*Lactobacillus paracasei*	Thermal	80 °C/20 min	UVB-induced skin cells (NHDF and B16F10)	Reduced DNA damage in NHDF and B16F10 cells, increased GSH content, antioxidant enzyme activity, and mRNA levels to alleviate UVB-induced oxidative damage, reduced UVB-induced photoaging in NHDF cells.	[109]
*Lactiplantibacillus plantarum*	Thermal	100 °C/20 min	Mouse macrophages	Complete cell wall of *L. plantarum* induced IL-12 secretion via actin-dependent phagocytosis, modulating immune responses.	[33]
*Lactobacillus plantarum*	Thermal	80 °C/10 min	Loperamide-induced constipated rats	Improved fecal pellet count, weight, water content, and intestinal contractility, increased mucosal layer thickness and goblet cell count, downregulated inflammatory cytokine levels.	[110]
*Lactobacillus paracasei* 6-1	Low temperature	65 °C/30 min	RAW 264.7 macrophages	Downregulated pro-inflammatory cytokines (TNF-α, IL-11, IL-6, IL-12), upregulated anti-inflammatory cytokine IL-10, repaired oxidative damage in colitis.	[111]
*Lactobacillus casei* DKGF7	Thermal	121 °C/15 min	IBS model rats	Reduced serum corticosterone levels, lowers colonic inflammatory cytokines, and increased tight junction protein expression.	[112]
*Lactobacillus plantarum* H-6	Thermal	90 °C water bath/30 min	Hypercholesterolemic mice	Reduced serum and liver lipid levels, improved glucose tolerance and insulin sensitivity, modulated gut microbiota.	[113]
*Lactobacillus plantarum* GMNL-6 and *Lactobacillus paracasei* GMNL-653	Thermal	121 °C/30 min	Tail-injured mice	Promoted wound healing, reduced fibrosis, and exhibited anti-fibrotic effects.	[114]
*Lactobacillus gasseri* CP2305	Pasteurization	Below 90 °C	Male/female volunteers aged 20–70	Increased defecation frequency was observed, accompanied by elevated abundance of beneficial gut microbiota and enhanced total power of autonomic nervous activity.	[115]
*Lactobacillus paracasei* MCC1849	Thermal	Pasteurization in hot water	241 adult healthy volunteers	The incidence of common colds, total symptom days, and symptom scores showed significant improvement, while stress-induced emotional deterioration was less pronounced.	[116]
*Lactobacillus gasseri* LA806	Thermal	70 °C/10 min	Bovine mammary epithelial Cells (bMEC)	Downregulated pro-inflammatory cytokine expression and demonstrated barrier-enhancing and immunomodulatory properties that prevented Staphylococcus aureus colonization in bovine mammary glands.	[117]
*Lactobacillus rhamnosus* GG MTCC 1048	Thermal	80 °C/20 min (Water bath)	LACA mice	Reduced the severity and duration of infection with *Giardiasis*, while elevating anti-Giardia IgA antibodies and nitric oxide levels in serum and intestinal fluid.	[118]
*Bifidobacterium longum* BR-108	High pressure and thermal	105 °C/20 min	Male obese TSOD mice	Significantly reduced body weight and blood sugar levels, while lowering levels of cholesterol, triglycerides, and NEFA. Serum and urine creatinine levels also decreased.	[119]
*Lacticaseibacillus casei* 01	Ohmic Heating	8 V/cm 95 °C/7 min, 60 Hz	15 healthy subjects	Inhibited α-glucosidase and α-amylase activity, reduced blood glucose response.	[120]
*Bifidobacterium bifidum* B1628	Thermal	90 °C/15 min	Colitis induced by DSS in mice	Alleviated inflammatory severity and tissue damage while improving DSS-induced gut dysbiosis and remodeling intestinal microbiota composition.	[121]

**Table 3:** Examples of non-thermal inactivation of postbiotics.

Strain	Inactivation Method	Conditions	Target	Function	Reference
*Lactobacillus plantarum* A3, *Lactobacillus reuteri* DMSZ 8533, and *Lactobacillus acidophilus* CICC 6074	Ultrasonic	Ice water bath ultrasonic for 30 min	LPS-induced RAW 264.7 macrophages and Caco-2 cells	LTA from the three strains significantly reduced inflammation, decreased TNF-α, IL-6, and IL-10 levels; LTA from *L. reuteri* DMSZ 8533 blocked LPS-triggered MAPK and NF-κB pathway expression.	[135]
*Bifidobacterium animalis* Bb-12	Gamma irradiation	The cobalt-60 multifunctional irradiator is irradiated at 2.5 KGy	Male Wistar rats	Serum glucose and total cholesterol levels were reduced. The abundances of *Firmicutes* and *Actinomycetes* increased, while the abundances of *Bacteroids* decreased.	[136]
*Lactobacillus* *plantarum* L12, *Lactobacillus* *reuteri* DSM 20016, *Bifidobacterium longum* Bb46, *Bifidobacterium infantis* Bb02	Ultrasonic	130 W, 20 kHz, net power: 40, 60, and 80%, duration: 2, 4, and 6 min, pulse set to 2 s	Caco-2 cell	The adhesion of *Lactobacillus reuteri* DSM 20016 to Caco-2 cells was enhanced.	[137]
*Lacticaseibacillus casei* 01	Ultrasonic	20 kHz, 40 min, 792 W	Male obese rat	Lower cholesterol levels and control insulin resistance in obese rats. Increased beneficial bacteria and reduced harmful bacteria in the gut.	[138]
*Bifidobacterium animalis* subsp. lactis (*B. lactis*)	Supercritical carbon dioxide	10 MPa, 40 °C, 180 min	Wistar male rat	Decreased serum total cholesterol level. Increased serum albumin and creatinine levels and decrease HDL-cholesterol levels.	[139]
*Lacticaseibacillus casei* subsp. paracasei 1 (*L. casei*)	Irradiation	Cobalt-60 multifunctional irradiator, dose: 2.5 KGy, 60 min	Wistar male rat	Decreased serum total cholesterol level.	[139]

## Data Availability

The data presented in this study are available on request from the corresponding author.
